# The Association Between Pressure Ulcer Development and In‐Hospital Outcomes: A Nationwide Hospitalization‐Level Study in Korea

**DOI:** 10.1111/jan.70678

**Published:** 2026-07-16

**Authors:** Seohyun Woo, Seungju Kim, Ji Eun Kim, Sun Jung Kim

**Affiliations:** ^1^ Department of Medical Science Soonchunhyang University Graduate School Asan‐Si Republic of Korea; ^2^ Department of Health System, College of Nursing The Catholic University of Korea Seoul Republic of Korea; ^3^ Department of Health Administration and Management College of Medical Science, Soonchunhyang University Asan‐Si Republic of Korea

**Keywords:** healthcare costs, in‐hospital mortality, length of stay, nurse staffing, pressure ulcer

## Abstract

**Aims:**

To estimate the nationwide incidence of pressure ulcers among hospitalized patients in Korea and to examine their associations with in‐hospital mortality, length of stay, and total inpatient costs.

**Design:**

A cross‐sectional study using retrospective cohort data.

**Methods:**

We analysed hospitalization‐level data using a nationally representative sample cohort from the National Health Insurance database. Pressure ulcers were identified using secondary diagnosis codes to approximate hospital‐acquired cases. Multivariable regression models were used to examine factors associated with pressure ulcer development, in‐hospital mortality, length of stay, and total inpatient costs.

**Data Sources:**

National Health Insurance sample cohort data from 2016 to 2019.

**Results:**

The incidence of pressure ulcers among hospitalized patients was low but showed clear associations with adverse outcomes. Older age, severe disability, medical aid coverage, admission to long‐term care hospitals, and lower nurse staffing levels were associated with a higher likelihood of pressure ulcer development. Pressure ulcers were also associated with increased in‐hospital mortality, longer hospital stays, and higher total inpatient costs.

**Conclusion:**

Pressure ulcers are associated with substantial clinical and economic burden and may serve as indicators of patient vulnerability and healthcare quality. Strengthening preventive strategies may improve patient outcomes and reduce avoidable healthcare utilization.

**Implications for the Profession and/or Patient Care:**

Pressure ulcers represent not only a clinical complication but also a marker of healthcare quality at the organizational level. Adequate nurse staffing and strengthened preventive care processes are essential to reduce pressure ulcer risk, particularly among high‐risk populations such as older adults and patients in long‐term care hospitals.

**Patient of Public Contribution:**

This study used de‐identified secondary data from a national database, and therefore no patients, service users, care‐givers, or members of the public were involved in the design, conduct, analysis, or interpretation of the study.

## Impact

- What problem did the study address?
  ○ This study addressed the lack of nationwide evidence on the incidence and impact of pressure ulcers among hospitalized patients in Korea.
- What were the main findings?
  ○ Pressure ulcers were associated with higher in‐hospital mortality, longer length of stay, and increased healthcare costs and were more likely to occur in vulnerable patient groups and under lower nurse staffing conditions.
- Where and on whom will the research have an impact?
  ○ The findings will have complications for nursing management, hospital administrators, and policymakers, particularly in settings caring for older adults and patients in long‐term hospitals.

## 1 Introduction

Patients have a fundamental right to receive safe care in a safe environment (Kim et al. 2007). However, the healthcare environment became more complex due to advances in medicine and technology, exposing patients to more threats to their safety (Kim, Hwang, et al. 2017). Consequently, the Institute of Medicine has identified patient safety as a key area of healthcare quality and one of the primary goals of healthcare (Donaldson et al. 2000). In addition, the US Agency for Healthcare Research and Quality (AHRQ) reports that 4%–16% of hospitalized patients experience adverse events during hospitalization in its adopted safety indicators (Patient Safety Indicators 2007), and in Korea, the trend of domestic and international interest in statistics associated with medical institution accreditation, medical errors, and patient safety indicators is increasing (Nam and Lim 2012).

Intrinsically, patient safety is an important area that can threaten the health of all citizens worldwide, and the World Health Organization (WHO) has identified the importance and seriousness of patient safety‐related issues and adopted a resolution at the WHO General Assembly in 2002, and Korea has also enacted the Patient Safety Act in 2015 (Seo 2016). Patient safety incidents are errors occurring during the hospital care process and include adverse drug events, blood transfusions, surgical injuries, wrong‐site surgery, falls, and pressure ulcers (Kim, Hwang, et al. 2017).

## 2 Background

Pressure ulcers are a type of injury characterized by damaged primary defence of the skin by repeated or sustained pressure and friction applied to a specific area of the body, resulting in necrosis of the skin or tissue underneath, and are currently occurring in various diseases (Ratliff and Rodeheaver 1999; Yang and Kim 1998; Cho et al. 2005). This disrupted primary defence of the skin provides an opportunity for bacterial infection, which can lead to bone and muscular infections, delayed recovery, and life‐threatening complications (Lee 2003). Risk factors for pressure ulcer occurrence include a patient's health status, mobility level, and nutritional deficiencies, as well as physical factors such as pressure, moisture, and friction (Baumgarten et al. 2008). Pressure ulcers are a recent patient safety indicator designated by the AHRQ, with approximately 3 million people in the USA treated (Guralnik et al. 1988) and 60,000 patients dying from their complications and experiencing reduced quality of life due to pain, infection, and prolonged hospitalization (Raleigh et al. 2008). Previous studies (Reid and Morison 1994) have reported that pressure ulcers occur in 3%–10% of all hospitalized patients, with incidence particularly high in chronic neurological and intensive care unit patients (Daechsel and Conine 1985; Bergstrom et al. 1987). These pressure ulcers result in physical and psychological disabilities for the affected individuals and increase healthcare costs at both individual and national levels (Yun and Park 2020). Previous studies (Clay 2000; Erwin‐Toth 1995) have demonstrated that pressure ulcers cost £60–200 million per year in the United Kingdom and approximately $1.3 billion in the USA for pressure ulcer treatment over 1 year, and pressure ulcers have been associated with an additional 8.2 days of hospitalization (Allman et al. 1999). Despite the evident seriousness of pressure ulcers, the number of pressure ulcers is continuously increasing (Nam and Lim 2012), indicating gaps in pressure ulcer management. Furthermore, the implied importance of pressure ulcers in patient safety indicators is increasing (Song and Choi 1991). During these trends, previous studies (Jung 2011) have reported that although Korea is not currently a country with high national healthcare expenditure compared to Organization for Economic Cooperation and Development (OECD) countries, problems like those faced by other industrialized countries with high national healthcare expenditure are expected. Since Korea operates a healthcare market under a National Health Insurance system, its government should manage healthcare expenditures with timely and effective policies in preparation for the increasing burden on the national economy due to increasing healthcare expenditures (Jung 2011). However, in Korea, current studies only analysed the incidence of pressure ulcers and related factors in specific groups such as newborns or nursing home inpatients (Moon 2013; Choi and Ju 2012) and the length of stay due to pressure ulcers (Nam and Lim 2012); however, studies analysing the incidence of pressure ulcers and the length of stay due to the disease and the increased medical expenses using cohort data for the entire population are lacking.

## 3 The Study

### 3.1 Aim

Therefore, this study aimed to estimate the hospitalization‐level incidence of pressure ulcers in Korea using data from the Health Insurance sample cohort 2.22016–2019 to provide basis for policy suggestions for managing healthcare expenditures and patient safety based on the pressure ulcer incidence.

## 4 Methods

### 4.1 Data Collection

This study analysed the hospitalization‐level incidence of pressure ulcers in Korea using the Health Insurance Cohort 2.2 2016–2019 data. The sample cohort data are designed and extracted to be representative of the entire Korean population and are a random sample of National Health Insurance and medical benefit patients nationwide in 2002, categorized by gender, age, income quintile, and eligibility, and collected for 17 years from 2002 to 2019. The sample cohort data contain various information on patients; are extracted from the National Health Information Database (DB); are collected for managing, billing, and paying medical expenses for National Health Insurance and medical benefits; and consist of three DBs: eligibility, medical treatment, and medical examination (Kim, Kim, et al. 2017). A total of 64,050,150 inpatient and outpatient data were collected by merging eligibility and insurance tables, medical treatment tables, and nursing institution tables of 2016–2019 data. Of the raw data collected, 6,503,173 patients were finally extracted by limiting the data to hospitalized patients only. All analyses were conducted at the hospitalization level, such that multiple hospitalizations for the same individual were treated as separate observations.

### 4.2 Key Variables

The variables used in the study are described in detail in the sample cohort DB 2.2 User's Manual, and the variables used in this study are as follows:

1. The dependent variable was the occurrence of pressure ulcers. Consistent with previous studies (Pyo et al. 2024), pressure ulcers were identified using KCD code (‘L89’, ‘L89.0’, ‘L89.1’, ‘L89.2’, and ‘L89.9’) recorded as secondary diagnoses among hospitalized patients whose primary diagnosis was not a pressure ulcer. This restriction was applied to approximate hospital‐acquired pressure ulcers, in accordance with the Agency for Healthcare Research and Quality (AHRQ) Patients Safety Indicator (PSI) framework (Rivard et al. 2005). Because the PSI definition identifies pressure ulcers based on secondary diagnosis codes to distinguish conditions that arise during hospitalization rather than those present at admission, limiting cases to secondary diagnoses allowed us to more closely capture potential in‐hospital occurrences.
2. Control variables include the demographic and socioeconomic characteristics of hospitalized patients, disability status, surgery status, type of medical institution visited, type of insurance, comorbidity burden, nurse staffing level, primary diagnosis categories, and year of admission. Age was categorized as ‘under 60’, ‘60–69’, ‘70–79’, and ‘80 years or older’. Income levels were categorized according to health insurance premium quintiles as ‘0–4th’, ‘5th–7th’, ‘8th–10th’ quintiles. Disability status was categorized as ‘none’, ‘mild’, and ‘severe’. The type of medical institution visited was classified into ‘hospital’, ‘general hospital’, ‘tertiary hospital’, and ‘long‐term care hospital’. The insurance type was categorized as ‘local subscriber’, ‘employee’, and ‘medical aid beneficiary’ regardless of the dependency status. Comorbidity burden was measured using the Charlson Comorbidity Index (0,1,2, ≥ 3). Nurse staffing level was defined as the number of nurses per 100 beds and categorized into quartiles. To adjust for differences in diagnostic composition, primary diagnoses accounting for ≥ 6% of cases among patients with secondary pressure ulcer diagnoses were retained as separate categories, while the remaining diagnoses were grouped as ‘others’. Year of admission (2016–2019) was also included.

### 4.3 Statistical Analysis

The analytical methods for this study were as follows. Descriptive statistics were first used to summarize the distribution of primary diagnoses among hospitalizations with pressure ulcer development. Frequencies and percentages were calculated for all study variables. Differences in patient, clinical, and institutional characteristics between hospitalizations with and without pressure ulcers were assessed using Pearson's chi‐square tests.

To identify factors independently associated with pressure ulcer development, a multivariable logistic regression model was fitted with pressure ulcer occurrence (yes/no) as the dependent variable. Independent variables included demographic characteristics (sex, age, residential area, income level, and disability status), clinical characteristics (surgery status, Charlson Comorbidity Index [CCI], and primary diagnosis category), institutional characteristics (type of medical institution, insurance type, and nurse staffing level), and year of admission. Adjusted odds ratios (ORs) and 95% confidence intervals (CIs) were estimated.

To evaluate whether pressure ulcer development was independently associated with in‐hospital mortality, a separate multivariable logistic regression model was constructed with in‐hospital mortality as the dependent variable. Pressure ulcer status was included as the primary independent variable of interest, while all patient, clinical, institutional, and temporal covariates described above were included as adjustment variables. Results are presented as adjusted ORs and 95% CIs.

Healthcare resource utilization was assessed using length of stay (LOS) and total inpatient costs. To examine the independent association between pressure ulcer development and these outcomes, generalized linear models (GLMs) were performed with LOS and total inpatient costs as dependent variables. Pressure ulcer status was included as the primary explanatory variable, and the same covariates used in the logistic regression analyses were included for adjustment. Because LOS and cost data exhibited substantial right‐skewness, natural logarithmic transformations were applied prior to model estimation. Regression coefficients (estimates) and corresponding *p*‐values are reported.

All statistical analyses were conducted using SAS Enterprise Guide version 7.1 (SAS Institute Inc., Cary, NC, USA). Statistical significance was defined as a two‐sided *p*‐value < 0.05.

### 4.4 Ethical Considerations

This study was approved by the Soonchunhyang University Institutional Review Board (SCH IRB) (Approved No. SCH IRB 2026‐03‐018) on March 25, 2026. The requirement for informed consent was waived due to the use of de‐identified secondary data.

## 5 Results

### 5.1 Characteristics of Patients by Pressure Ulcer Development

Table 1 shows the characteristics of inpatient admissions according to pressure ulcer development among 6,503,173 hospitalizations. Pressure ulcers occurred in 18,368 admissions (0.28%) and were more frequently observed in older age groups, particularly among admissions of patients aged ≥ 80 years (0.55%), as well as in females (0.30%) and those residing in non‐metropolitan areas (0.30%) (all *p* < 0.0001). A higher proportion of pressure ulcers was also noted in admissions involving severe disabilities (0.58%), medical aid beneficiaries (0.49%). By institution type, long‐term care hospitals showed the highest proportion of pressure ulcer‐related admissions (0.77%), and lower nursing staff levels were associated with a higher proportion of pressure ulcer occurrence (*p* < 0.0001).

**TABLE 1:** Characteristics of patients by pressure ulcer development.

Variables	Inpatient (*N* = 6,503,173)				
	Pressure ulcer: No (*N* = 6,484,805)		Pressure ulcer: Yes (*N* = 18,368)		*p*
	*N*	%	*N*	%	
Gender					
Male	2,929,572	99.74	7565	0.26	< 0.0001
Female	3,555,233	99.70	10,803	0.30	
Age					
< 60	2,562,651	99.92	2173	0.08	< 0.0001
60–69	1,080,629	99.78	2384	0.22	
70–79	1,309,761	99.59	5347	0.41	
80 ≤	1,531,764	99.45	8464	0.55	
Patient residence area					
Metropolis	2,623,235	99.74	6792	0.26	< 0.0001
Other area	3861.570	99.70	11,576	0.30	
Income level by insurance premium					
0–4	2,525,385	99.69	7772	0.31	< 0.0001
5–7	1,534,470	99.80	3147	0.20	
8–10	2,424,950	99.69	7449	0.31	
Disability					
None	4,763,881	99.79	10,252	0.21	< 0.0001
Mild	790,577	99.66	2735	0.34	
Severe	930,347	99.42	5381	0.58	
Surgery					
No	5,047,483	99.68	15,973	0.32	< 0.0001
Yes	1,437,322	99.83	2395	0.17	
Type of institution					
Hospital	1,721,923	99.86	2387	0.14	< 0.0001
General hospital	2,155,227	99.89	2387	0.11	
Tertiary hospital	1,000,350	99.89	1073	0.11	
Long‐term care hospital	1,607,305	99.23	12,521	0.77	
Type of insurance					
Local	1,833,455	99.76	4485	0.24	< 0.0001
Employee	3,826,135	99.74	9782	0.26	
Medical aid	825,215	99.51	4101	0.49	
CCI					
0	3,442,762	99.71	9947	0.29	< 0.0001
1	1,157,224	99.79	2459	0.21	
2	762,420	99.72	2159	0.28	
3 ≤	1,122,399	99.66	3803	0.34	
Nursing staff per 100 beds (quartiles)					
Q1	761,759	99.42	4406	0.58	< 0.0001
Q2	947,407	99.27	6979	0.73	
Q3	1,116,647	99.72	3082	0.28	
Q4	3,658,992	99.89	3901	0.11	
Primary diagnosis					
A	275,839	99.53	1312	0.47	< 0.0001
C	603,654	99.79	1241	0.21	
F	646,327	99.39	3938	0.61	
G	538,291	99.50	2699	0.50	
I	781,016	99.77	1802	0.23	
J	727,006	99.71	2128	0.29	
Others	2,912,672	99.82	5248	0.18	
Year					
2016	1,524,808	99.70	4548	0.30	< 0.0001
2017	1,605,234	99.66	5433	0.34	
2018	1,669,639	99.76	3972	0.24	
2019	1,685,124	99.74	4415	0.26	

### 5.2 Pressure Ulcer Disease Group

As presented in Table 2, mental and behavioural disorders accounted for the largest proportion of hospital admissions with pressure ulcer development (F, 21.44%), followed by diseases of the nervous system (G, 14.69%) and diseases of the respiratory system (J, 11.59%). Admissions with circulatory system diseases (I) comprised 9.81% of pressure ulcer cases, while infectious and parasitic diseases (A, 7.14%) and neoplasms (C, 6.76%) were also commonly observed. The remaining diagnostic categories each accounted for less than 6% of pressure ulcer‐related admissions.

**TABLE 2:** Pressure ulcer disease groups.

Main diagnosis	# Of pressure ulcer (*N* = 18,368)	
	*N*	%
F (mental and behavioural disorders)	3938	21.44
G (diseases of the nervous system)	2699	14.69
J (diseases of the respiratory system)	2128	11.59
I (diseases of the circulatory system)	1802	9.81
A (certain infectious and parasitic disease)	1312	7.14
C (neoplasms)	1241	6.76
S (injury, poisoning and certain other consequences of external causes)	1037	5.65
N (diseases of the genitourinary system)	989	5.38
E (endocrine, nutritional and metabolic diseases)	860	4.68
M (diseases of the musculoskeletal system and connective tissue)	712	3.88
K (diseases of the digestive system)	392	2.13
L (diseases of the skin and subcutaneous tissue)	369	2.01
R (symptoms, signs and abnormal clinical and laboratory findings, NEC)	358	1.95
T (injury, poisoning and certain other consequences of external causes)	191	1.04
D (neoplasms & diseases of the blood and blood‐forming organs and certain disorders involving the immune mechanism)	111	0.60
Z (factors influencing health status and contact with health services)	105	0.57
U (codes for special purposes)	62	0.34
B (certain infectious and parasitic disease)	32	0.17
Q (congenital malformations, deformations and chromosomal abnormalities)	28	0.15
H (diseases of the eye, adnexa, ear and mastoid process)	2	0.01

### 5.3 Odds Ratio for Pressure Ulcer Development by Different Patients' Characteristics

Table 3 presents the results of the multivariable logistic regression analysis for pressure ulcer development. Male was associated with higher odds of pressure ulcer development (OR 1.112, 95% CI: 1.078–1.147), and the odds increased substantially with age, with admissions of patients aged ≥ 80 years showing more than threefold higher odds compared with those aged < 60 years (OR 3.076). Admissions to long‐term care hospitals had markedly higher odds of pressure ulcer development than hospitals (OR 3.253). Severe disability (OR 1.931), medical aid coverage (OR 1.559), and lower nursing staff levels‐particularly in the second‐lowest quartile (OR 2.012)‐were also associated with increased odds. Among primary diagnosis, infectious and parasitic diseases (A, OR 2.252) and respiratory diseases (J, OR 1.445) showed higher odds, while the odds of pressure ulcer development decreased over time (OR 0.875).

**TABLE 3:** Odds ratio for pressure ulcer development by different patients' characteristics.

Variables	Pressure ulcer	
	Odds ratio	95% CI
Gender		
Male	1.112	1.078–1.147
Female	Ref.	—
Age		
< 60	Ref.	—
60–69	1.906	1.796–2.023
70–79	2.879	2.730–3.036
80 ≤	3.076	2.917–3.244
Patient residence area		
Metropolis	Ref.	—
Other area	1.130	1.097–1.165
Income level by insurance premium		
0–4	0.822	0.789–0.856
5–7	0.870	0.833–0.907
8–10	Ref.	—
Disability		
None	Ref.	—
Mild	1.094	1.048–1.142
Severe	1.931	1.863–2.003
Surgery		
No	Ref.	—
Yes	1.574	1.497–1.656
Type of institution		
Hospital	Ref.	—
General hospital	0.857	0.798–0.920
Tertiary hospital	0.907	0.830–0.992
Long‐term care hospital	3.253	3.079–3.437
Type of insurance		
Local	1.035	0.998–1.074
Employee	Ref.	—
Medical aid	1.559	1.485–1.636
CCI		
0	Ref.	—
1	1.116	1.067–1.169
2	1.245	1.187–1.307
3 ≤	1.334	1.281–1.388
Nursing staff per 100 beds (quartiles)		
Q1	1.649	1.526–1.782
Q2	2.012	1.866–2.169
Q3	1.271	1.183–1.366
Q4	Ref.	—
Primary diagnosis		
A	2.252	2.117–2.396
C	0.944	0.886–1.007
F	0.966	0.922–1.013
G	0.938	0.892–0.987
I	0.531	0.502–0.561
J	1.445	1.372–1.522
Others	Ref.	—
Year	0.875	0.863–0.887

### 5.4 Odds Ratio of In‐Hospital Mortality Associated With Pressure Ulcers

Table 4 shows the association between pressure ulcers and in‐hospital mortality based on multivariable logistic regression analysis. Admissions with pressure ulcers had substantially higher odds of in‐hospital mortality compared with those without pressure ulcers (OR 2.718, 95% CI: 2.557–2.889). Mortality odds increased sharply with age, with the highest odds observed among admissions of patients aged ≥ 80 years (OR 5.483). Male (OR 1.418), surgical admissions (OR 2.714), lower nursing staff levels (OR 1.643 for Q2), and high‐prevalence primary diagnosis categories (≥ 6% of cases), including neoplasms (C, OR 6.325) and respiratory diseases (J, OR 4.533)‐were associated with increased mortality. Mortality odds slightly decreased over time (OR 0.978).

**TABLE 4:** Odds ratio of in‐hospital mortality associated with pressure ulcers.

Variables	Pressure ulcer	
	Odds ratio	95% CI
Pressure ulcer		
Yes	2.718	2.557–2.889
No	Ref.	—
Gender		
Male	1.418	1.402–1.434
Female	Ref.	—
Age		
< 60	Ref.	—
60–69	1.786	1.749–1.823
70–79	2.882	2.829–2.936
80 ≤	5.483	5.386–5.580
Patient residence area		
Metropolis	Ref.	—
Other Area	0.950	0.939–0.961
Income level by insurance premium		
0–4	1.061	1.046–1.077
5–7	1.072	1.056–1.088
8–10	Ref.	—
Disability		
None	Ref.	—
Mild	0.927	0.912–0.942
Severe	1.129	1.110–1.148
Surgery		
No	Ref.	—
Yes	2.714	2.678–2.750
Type of institution		
Hospital	Ref.	—
General hospital	2.435	2.376–2.495
Tertiary hospital	2.381	2.317–2.447
Long‐term care hospital	1.545	1.508–1.583
Type of insurance		
Local	1.111	1.096–1.125
Employee	Ref.	—
Medical aid	1.151	1.129–1.175
CCI		
0	Ref.	—
1	0.995	0.978–1.011
2	1.100	1.082–1.120
3 ≤	1.186	1.169–1.203
Nursing staff per 100 bed (quartiles)		
Q1	1.482	1.436–1.529
Q2	1.643	1.595–1.692
Q3	1.244	1.215–1.274
Q4	Ref.	—
Primary diagnosis		
A	4.577	4.466–4.691
C	6.325	6.219–6.432
F	0.967	0.939–0.996
G	1.009	0.977–1.042
I	2.318	2.274–2.363
J	4.533	4.452–4.616
Others	Ref.	—
Year	0.978	0.973–0.983

### 5.5 Inpatient Length of Stay and Total Inpatient Charges by Pressure Ulcer Development

As reported in Table 5, admissions with pressure ulcers were associated with longer hospital stays and higher total inpatient charges compared with those without pressure ulcers (19.89 vs. 13.41 days; KRW 2,732,792 vs. KRW 2,628,889). This pattern was generally observed across most patient and institutional subgroups. Differences in length of stay and charges were evident among both surgical and non‐surgical admissions and across hospital types, although the magnitude of differences varied.

**TABLE 5:** Inpatient length of stay and total inpatient charges by pressure ulcer development.

Variables	Length of stay		Total cost of care (KRW)	
	Pressure ulcer		Pressure ulcer	
	Yes	No	Yes	No
	19.89	13.41	2,732,792	2,628,889
Gender				
Male	18.83	12.46	3,070,097	2,802,546
Female	20.63	14.18	2,496,588	2,485,792
Age				
< 60	18.25	9.07	2,977,357	2,123,722
60–69	21.20	12.89	3,721,039	3,013,893
70–79	20.14	15.44	2,993,145	3,208,059
80 ≤	19.78	19.29	2,227,177	2,707,194
Patient residence area				
Metropolis	18.16	13.35	2,822,661	2,704,279
Other Area	20.90	13.44	2,680,063	2,577,675
Income level by insurance premium				
0–4	18.96	14.34	2,721,304	2,555,263
5–7	21.25	12.01	2,947,091	2,571,391
8–10	20.28	13.31	2,654,244	2,741,948
Disability				
None	19.85	11.79	2,571,737	2,468,784
Mild	20.00	16.63	2,684,045	3,046,564
Severe	19.91	18.95	3,064,416	3,093,785
Surgery				
No	19.49	13.49	2,056,860	1,776,577
Yes	22.54	13.12	7,240,795	5,621,976
Type of institution				
Hospital	19.03	10.93	3,034,538	1,650,755
General hospital	18.62	10.13	5,027,953	2,952,556
Tertiary hospital	18.60	8.85	8,339,747	4,718,238
Long‐term care hospital	20.40	23.29	1,757,225	1,942,407
Type of insurance				
Local	19.37	13.49	2,855,245	2,705,763
Employee	20.60	12.62	2,798,971	2,632,899
Medical aid	18.74	16.85	2,441,019	2,439,499
CCI				
0	20.72	14.49	2,425,168	2,342,143
1	18.51	11.09	2,528,013	2,544,623
2	19.10	12.60	3,307,524	3,052,585
3	19.04	13.01	3,343,534	3,307,504
Nursing staff per 100 beds (quartile)				
Q1	20.33	20.89	1,721,137	1,765,873
Q2	20.47	20.68	1,841,653	1,839,209
Q3	20.28	14.38	2,368,413	1,853,999
Q4	18.03	9.67	5,757,563	3,249,507
Primary diagnosis				
A	11.91	7.74	2,165,983	1,764,180
C	16.63	11.91	3,296,173	3,962,059
F	24.05	25.20	1,917,138	1,953,845
G	23.77	20.84	2,550,183	2,579,683
I	22.25	17.34	3,077,786	3,798,797
J	15.76	8.92	3,121,922	1,989,856
Others	18.39	10.33	3,170,989	2,439,165
Year				
2016	20.00	13.57	2,321,306	2,367,783
2017	20.10	13.45	2387,478	2,491,946
2018	19.91	13.33	3,106,432	2,696,761
2019	19.49	13.29	3,245,461	2,928,355

### 5.6 Results of the GLM: Length of Stay and Total Cost of Care by Pressure Ulcer Development

As detailed in Table 6, generalized linear models with log‐transformed outcomes showed that pressure ulcer development was independently associated with longer length of stay (estimate = 0.016) and higher total inpatient costs (estimate = 0.082). Increasing age and surgical admissions were strongly associated with longer stays and higher costs, while lower nursing staff levels were consistently associated with increased length of stay and costs. Admissions to long‐term care hospitals showed longer stays and higher costs, and length of stay decreased over time whereas total costs increased.

**TABLE 6:** Results of the GLM: Length of stay and total cost of care by pressure ulcer development.

Variables	Length of stay		Total cost of care	
	Estimate	*p*	Estimate	*p*
Pressure ulcer				
Yes	0.016	0.0103	0.082	< 0.0001
No	Ref.	—	Ref.	—
Gender				
Male	−0.044	< 0.0001	−0.031	< 0.0001
Female	Ref.	—	Ref.	—
Age				
< 60	Ref.	—	Ref.	—
60–69	0.189	< 0.0001	0.276	< 0.0001
70–79	0.306	< 0.0001	0.424	< 0.0001
80 ≤	0.377	< 0.0001	0.505	< 0.0001
Patient residence area				
Metropolis	Ref.	—	Ref.	—
Other areas	0.002	0.0003	−0.0019	0.0667
Income level by insurance premium				
0–4	0.040	< 0.0001	0.054	< 0.0001
5–7	0.016	< 0.0001	0.020	< 0.0001
8–10	Ref.	—	Ref.	—
Disability				
None	Ref.	—	Ref.	—
Mild	0.143	< 0.0001	0.156	< 0.0001
Severe	0.204	< 0.0001	0.286	< 0.0001
Surgery				
No	Ref.	—	Ref.	—
Yes	0.526	< 0.0001	1.433	< 0.0001
Type of institution				
Hospital	Ref.	—	Ref.	—
General hospital	−0.007	< 0.0001	0.437	< 0.0001
Tertiary hospital	−0.198	< 0.0001	0.529	< 0.0001
Long‐term care hospital	0.420	< 0.0001	0.213	< 0.0001
Type of insurance				
Local	0.057	< 0.0001	0.063	< 0.0001
Employee	Ref.	—	Ref.	—
Medical aid	0.043	< 0.0001	−0.010	< 0.0001
CCI				
0	Ref.	—	Ref.	—
1	−0.053	< 0.0001	−0.029	< 0.0001
2	−0.032	< 0.0001	0.026	< 0.0001
3	−0.036	< 0.0001	0.033	< 0.0001
Nursing staff per 100 beds (quartile)				
Q1	0.293	< 0.0001	0.098	< 0.0001
Q2	0.250	< 0.0001	0.101	< 0.0001
Q3	0.145	< 0.0001	0.022	< 0.0001
Q4	Ref.	—	Ref.	—
Primary diagnosis				
A	−0.088	< 0.0001	0.023	< 0.0001
C	0.193	< 0.0001	0.370	< 0.0001
F	0.647	< 0.0001	0.654	< 0.0001
G	0.413	< 0.0001	0.578	< 0.0001
I	0.285	< 0.0001	0.587	< 0.0001
J	0.077	< 0.0001	0.213	< 0.0001
Others	Ref.	—	Ref.	—
Year	−0.023	< 0.0001	0.050	< 0.0001

## 6 Discussion

This nationwide cohort study examined the hospitalization‐level incidence of pressure ulcers among hospitalized patients in Korea. It also evaluated association with in‐hospital mortality, length of stay, and total inpatient costs. Several important findings emerged. First, pressure ulcers were associated with a significantly higher risk of in‐hospital mortality. Second, pressure ulcer development was linked to prolonged hospitalization and increased medical expenditure, even after adjusting for demographic, clinical, and institutional factors. Third, lower nurse staffing levels were consistently associated with higher odds of pressure ulcer development, higher mortality, and increased resource utilization. Together, these findings suggest that pressure ulcers may function not only as a clinical complication but also as a marker of patient vulnerability and healthcare quality.

The overall incidence of pressure ulcers in this study was 0.28% among hospitalized admissions. This proportion appears lower than rates reported in other studies, likely due to our stricter definition of using secondary diagnoses to approximate hospital‐acquired pressure ulcers (Kim et al. 2019). Pressure ulcers were more frequently observed among older patients, those with severe disabilities, medical aid beneficiaries, and those admitted to long‐term care hospitals. Advanced age is a well‐established risk factor for pressure ulcer development due to reduced mobility and delayed wound healing (Li et al. 2026; Hajhosseini et al. 2020). Similarly, patients with severe disabilities may experience limited mobility and impaired sensation, increasing susceptibility to sustained pressure and tissue injury (Sprigle et al. 2020). Comorbidity burden, as measured by the Charlson Comorbidity Index, was also associated with pressure ulcer development. This finding supports previous evidence that patients with multiple chronic conditions are more vulnerable to complications during hospitalization (Jaul et al. 2018). Among primary diagnostic categories with ≥ 6% prevalence in pressure ulcer cases, statistically significant associations were observed only for infectious diseases (A) and respiratory diseases (J). This suggests that pressure ulcer development may reflect overall clinical instability rather than diagnosis‐specific risk (Jaul et al. 2018).

One of the most clinically meaningful findings was the strong association between pressure ulcers and in‐hospital mortality (OR 2.718). Although causal inference cannot be established, pressure ulcers may serve as a marker of frailty, disease severity, and compromised physiological reserve (Allman et al. 1999). The occurrence of a pressure ulcer may reflect prolonged immobility, systemic inflammation, nutritional deficiencies, or overall deterioration in clinical status, all of which are associated with adverse outcomes. Therefore, pressure ulcer development during hospitalization may indicate heightened vulnerability requiring intensified monitoring and management.

Pressure ulcer development was associated with a 1.6% increase in length of stay and an 8.5% increase in total inpatient costs in log‐transformed models. Although the percentage increase in length of stay seems modest, absolute differences indicate a substantial population‐level burden (Hasan et al. 2025). Given the large number of hospitalizations annually, even small percentage increases may translate into considerable cumulative healthcare expenditures. Previous studies have similarly reported prolonged hospitalization and increased costs associated with pressure ulcers (Padula and Delarmente 2019). In aging societies such as Korea, where healthcare expenditure is steadily rising, preventable complications that increase length of stay and costs represent a significant concern for health system sustainability (Moon et al. 2022).

Lower nurse staffing quartiles were associated with higher odds of pressure ulcer development, in‐hospital mortality, and increased length of stay and costs. Adequate nurse staffing is critical for patient safety and quality of care (Aiken et al. 2012). Nurses play a central role in repositioning patients, assessing skin, managing moisture and nutrition, and implementing preventive protocols (Kim et al. 2022). Insufficient staffing may reduce the capacity to deliver timely preventive care, thereby increasing the risk of avoidable complications. Although causality cannot be directly inferred, the consistent pattern observed in this study suggests that nurse staffing is a modifiable institutional factor that may contribute to improved patient outcomes.

Admissions to long‐term care hospitals showed markedly higher odds of pressure ulcer development. These institutions often care for older adults with functional impairment and multiple chronic conditions, which may partially explain the elevated risk. This finding underscores the need for strengthened preventive strategies in settings where high‐risk patients are concentrated. Targeted interventions—such as standardized risk assessment tools, early mobilization protocols, and routine skin surveillance—may be particularly beneficial in these environments (Tayyib et al. 2015).

Interestingly, the odds of pressure ulcer development decreased over time, whereas total inpatient costs increased (Choragudi et al. 2024). This pattern may reflect gradual improvements in prevention and documentation practices alongside broader trends of rising medical expenditure. Continued monitoring of pressure ulcer incidence is necessary to evaluate the effectiveness of patient safety policies implemented under the Korean National Health Insurance system.

### 6.1 Limitations

This study has several limitations that should be considered. First, causal relationships cannot be established due to observational design. Second, POA indicators were unavailable, so pressure ulcers were identified using secondary diagnoses, potentially misclassifying pre‐existing ulcers. Third, ulcer severity was not captured, limiting detailed clinical interpretation. Fourth, primary diagnosis categories may not fully capture clinical heterogeneity. Fifth, nurse staffing level is an institutional‐level indicator and may not reflect bedside staffing variations. Sixth, mild or non‐coded pressure ulcers and non‐covered expenditures were not captured, possibly underestimating clinical and economic burden.

However, this study is significant for several reasons. First, it is the first nationwide analysis to report the incidence of pressure ulcers in Korea using a representative sample cohort of the National Health Insurance, providing a comprehensive estimate of the burden of this patient safety issue. Second, by linking pressure ulcer occurrence to in‐hospital mortality, length of stay, and inpatient costs, the study demonstrates the substantial clinical and economic impact of this preventable complication. Third, this study highlights modifiable institutional factors, such as nurse staffing levels, that may influence patient outcomes, offering actionable insights for hospital management and policy makers. Finally, the findings underscore the importance of prioritizing pressure ulcer prevention in the context of a rapidly aging population and rising healthcare expenditures, suggesting that targeted interventions could improve patient safety while contributing to more sustainable use of healthcare resources.

## 7 Conclusion

This nationwide cohort study demonstrated that pressure ulcer development during hospitalization is independently associated with higher in‐hospital mortality, longer length of stay, and increased inpatient costs. These findings suggest that pressure ulcers represent not merely a localized skin condition but a clinically meaningful indicator of patient vulnerability and adverse hospital outcomes. Lower nurse staffing levels were consistently associated with a higher likelihood of pressure ulcer development and poorer clinical and economic outcomes. Adequate nurse staffing is a potentially modifiable factor that may improve patient safety and resource utilization (Alanazi et al. 2023). Taken together, these findings highlight the importance of strengthening preventive strategies for pressure ulcers at both institutional and policy levels. Efforts such as systematic risk assessment, early intervention, and appropriate workforce allocation may improve patient outcomes while mitigating economic burden.

## Author Contributions

Seohyun Woo conducted all statistical analyses and prepared the first draft of the manuscript. Seungju Kim contributed to the interpretation of the data and critically revised the manuscript. Ji Eun Kim contributed to the critical revision of the manuscript. Sun Jung Kim conceived and designed the study, supervised the data analysis and the overall research process, and critically revised the manuscript. All authors reviewed and approved the final version of the manuscript.

## Funding

This work was supported by the Soonchunhyang University Research Fund.

## Ethics Statement

This study was approved by the Soonchunhyang University Institutional Review Board (SCH IRB) (Approved No. SCH IRB 2026‐03‐018) on March 25, 2026.

## Consent

The requirement for informed consent was waived due to the use of de‐identified secondary data.

## Conflicts of Interest

The authors declare no conflicts of interest.

## Supporting information


**Data S1:** STROBE statement—checklist of items that should be included in reports of cross‐sectional studies.

## Data Availability

The data that support the findings of this study are available on request from the corresponding author. The data are not publicly available due to privacy or ethical restrictions.
